# The Act of Controlling Adult Stem Cell Dynamics: Insights from Animal Models

**DOI:** 10.3390/biom11050667

**Published:** 2021-04-30

**Authors:** Meera Krishnan, Sahil Kumar, Luis Johnson Kangale, Eric Ghigo, Prasad Abnave

**Affiliations:** 1 Regional Centre for Biotechnology, NCR Biotech Science Cluster, 3rd Milestone, Gurgaon-Faridabad Ex-pressway, Faridabad 121001, India; meera.krishnan@rcb.res.in (M.K.); sahil.kumar@rcb.res.in (S.K.); 2 IRD, AP-HM, SSA, VITROME, Aix-Marseille University, 13385 Marseille, France; kangaleluis@yahoo.fr; 3 Institut Hospitalo Universitaire Méditerranée Infection, 13385 Marseille, France; eric.ghigo@gmail.com; 4 TechnoJouvence, 13385 Marseille, France

**Keywords:** adult stem cell, signaling pathway, animal model, intestinal stem cell, neural stem cell, hematopoietic stem cell, epidermal stem cell

## Abstract

Adult stem cells (ASCs) are the undifferentiated cells that possess self-renewal and differentiation abilities. They are present in all major organ systems of the body and are uniquely reserved there during development for tissue maintenance during homeostasis, injury, and infection. They do so by promptly modulating the dynamics of proliferation, differentiation, survival, and migration. Any imbalance in these processes may result in regeneration failure or developing cancer. Hence, the dynamics of these various behaviors of ASCs need to always be precisely controlled. Several genetic and epigenetic factors have been demonstrated to be involved in tightly regulating the proliferation, differentiation, and self-renewal of ASCs. Understanding these mechanisms is of great importance, given the role of stem cells in regenerative medicine. Investigations on various animal models have played a significant part in enriching our knowledge and giving In Vivo in-sight into such ASCs regulatory mechanisms. In this review, we have discussed the recent In Vivo studies demonstrating the role of various genetic factors in regulating dynamics of different ASCs viz. intestinal stem cells (ISCs), neural stem cells (NSCs), hematopoietic stem cells (HSCs), and epidermal stem cells (Ep-SCs).

## 1. Introduction

The sustained functions of various tissues in the body are maintained by a population of cells with self-renewal ability called adult stem cells (ASCs) or tissue-resident stem cells. These cells are generally multipotent and differentiate into various effector cells, thus replenishing tissues like blood, skin, muscle, etc., throughout life. ASCs are usually more quiescent than their progenies, and they remain such unless the need arises for division or differentiation. Research over the years has identified tissue-resident cells with the varying capacity of self-renewal and differentiation. The identified ASCs so far include mesenchymal stem cells (MSCs), which give rise to osteogenic, adipogenic and chondrogenic lineages; hematopoietic stem cells (HSCs), which replenish cell types of the blood; germline stem cells giving rise to germ cells; epithelial stem cells of the skin, found on the bulge of hair follicles; neural stem cells in the central nervous system; muscle stem cells or satellite stem cells found usually in between the basal membrane and cell membrane of myofibers; stem/progenitor cells in the liver, lungs, cardiac tissue, pancreas etc. These identified adult stem cells could include “true” stem cells or a population of stem and progenitor cells. Research done by different groups has identified different subsets of such cells within the same tissue, making it difficult to give a consistent definition. 

When it comes to defining tissue-resident stem cells, the general consensus is their potential to self-renew and differentiate. Self-renewal may be defined as their potential to regenerate themselves during each division, and differentiation capacity is their ability to give rise to mature effector cells without losing the original stem cell pool. ASCs are classified as pluripotent, multipotent or unipotent based on their differentiation capacity. The self-renewal and multipotency of stem cells are found to diminish over time due to known and unknown genetic, epigenetic and environmental factors. Ageing is associated with this gradual reduction in the capacity of stem cells to replace old tissue. 

In the past couple of decades, there has been extensive research going on in the field of stem cell biology, primarily due to their potential in regenerative medicine and therapy. Even after decades of research, we still know very little about their true nature. This review aims to shed some light on adult stem cell dynamics based on In Vivo evidence from mainly four types of adult stem cells: intestinal stem cell (ISC), neural stem cells (NSC), hematopoietic stem cell (HSC) and epidermal stem cell (EpSC). The role of genetic factors is discussed here, emphasizing the insights gained from animal models.

## 2. Genetic Factors Regulating ASC Dynamics

The self-renewal and differentiation of ASCs are strictly regulated by an intricate network of cell intrinsic and extrinsic factors. The intrinsic factors regulating stem cells include transcription factors, cell signaling pathways, cel -cycle control, and metabolic pathways [1]. Some of the most studied stem cell regulation pathways are Wnt, TGF (transforming growth factor) -β, Notch, Hedgehog (Hg) and MAP (mitogen-activated protein) kinase pathway. These signaling pathways are also involved in the growth and differentiation of progenitors and other lineage-restricted cells. Different ASCs respond similarly to these intrinsic mechanisms indicating the similar underlying physiology of most stem cells, meaning that the differentiating factor among these cells might be their location and other extrinsic factors. 

### 2.1. Wnt Signaling

The Wnts are a family of secreted glycoproteins that bind to a seven trans-membrane receptor called Frizzled, which may be coupled to a G-protein. The pathway downstream has been found to diverge into at least three different branches, viz. the canonical β-catenin pathway and two non-canonical β-catenin independent pathways: JNK (Jun-N Terminal Kinase) pathway and Wnt-Calcium pathway [2]. Studies indicate that canonical Wnt signaling plays a major role in maintaining the stem cells in their undifferentiated self-renewing state. 

**Figure 1:**
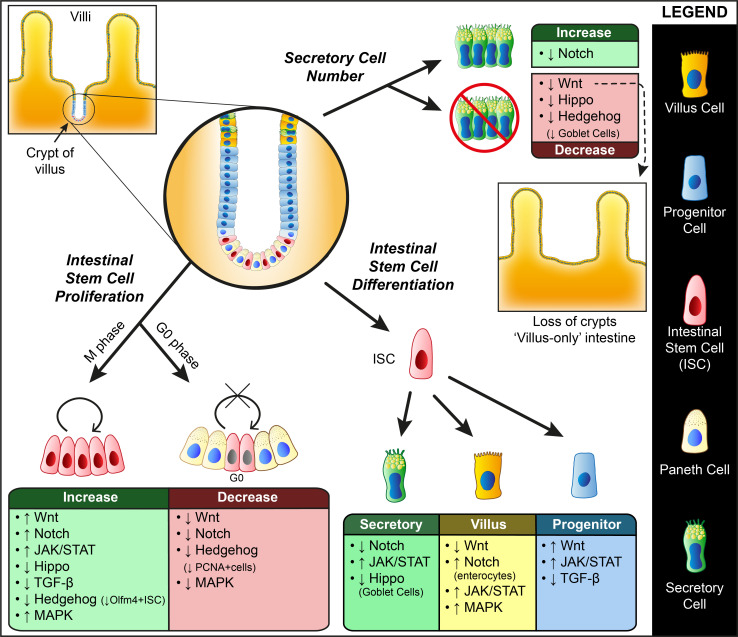
Genetic regulation of ISC (intestinal stem cell) dynamics. ISCs maintain all the differentiated cell types in the intestinal epithelium. The figure shows a magnified crypt morphology with a distribution of different cell types along the crypt and various signaling pathways modulating ISC dynamics. The direction of arrows near the pathways indicates upregulation or downregulation. In general, Wnt, Notch, JAK/STAT, and MAPK pathways are promoters of proliferation in ISCs, whereas TGF-β and Hippo are negative regulators. Loss of Wnt and hedgehog leads to differentiation defects with loss of crypt proliferation and loss of ISCs, although there is contradicting evidence where hedgehog increases proliferation. The notch is also necessary to maintain ISCs in their progenitor phenotype as loss of Notch was found to induce differentiation. JAK/STAT also plays a role in cell fate specification as loss of signaling causes defective differentiation and failure in specification.

ISC: The Wnt signaling cascade is one of the major controllers of division and differentiation and studies using In Vivo models have shown the immense role they play in ISCs (Figure 1). The first evidence showing the role of Wnt signaling in stem cell biology comes from ISCs, when TCF4 (Transcription factor 4), a downstream effector of β-catenin pathway disruption in mice, led to the loss of ISCs in 1998 [3]. Nuclear β-catenin, a major downstream effector of the canonical Wnt pathway, was found to accumulate at the stem/progenitor compartments at the bottom of adult intestinal crypts [4,5]. In studies using villin-Dkk1(Dickkopf related protein 1) transgenic mice that ectopically express Dkk1 (Wnt Inhibitor), it was found that Wnt inhibition significantly reduces epithelial proliferation, with loss of crypts and secretory lineages and disrupted intestinal homeostasis. They were able to establish a direct role of Wnt signaling in intestinal cell proliferation and secretory cell differentiation [6]. Much earlier studies in fetal mice deficient for TCF-4 have produced phenotypes showing a lack of proliferative intestinal compartments. This produced mice with intestines composed entirely of differentiated non-dividing villus cells [3,4]. Another study using AhCre transgenic mice has shown that loss of APC (adenomatous polyposis coli) causes accumulation of β-catenin, leading to activated Wnt signaling, which is also associated with perturbations in differentiation, migration, proliferation, and apoptosis. These APC-deficient cells were said to maintain a “crypt-progenitor cell” phenotype and results within days in the entire repopulation of villi by crypt-like cells [7]. Generally, in ISC, inhibition in the Wnt cascade seems to block proliferation and activation of the Wnt signal leads to induce proliferation with differentiation defects. In contrast, activation of Wnt signals in the intestine’s differentiated cells leads to massive expansion, eventually leading to cancer. 

*Drosophila* model system has also been used to study signaling cascades involved in ISC. Studies involving components of the Wnt signaling pathway like Wnt ligand Wingless (Wg), APC or shaggy (*Drosophila* equivalent of Glycogen Synthase Kinase (GSK) 3-β) have reported that ISCs defective for these components show a higher proliferation rate with disrupted homeostasis, hyperplasia, and multilayering of the midgut epithelium. Similarly, reduction in the ligand Wg results in ISC quiescence and differentiation, whereas its overexpression caused an increase in the number of ISCs. It has also been reported that other components of the pathway, like Frizzled, Dishevelled and Armadillo, are necessary for ISC self-renewal in an autonomous manner. There are also suggestions that the Notch pathway operates downstream of Wg and is essential for maintaining the balance between self-renewal and differentiation. The effects of Wnt signaling in *Drosophila* ISCs are relatively mild compared to other signaling networks. For instance, APC is required for regulating stem cell proliferation but is not necessary for self-renewal or fate specification [8,9,10]. 

**Figure 2:**
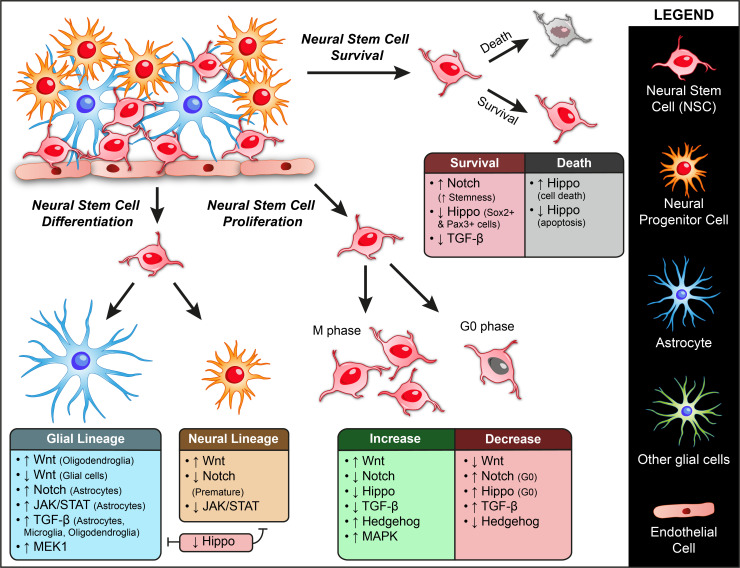
Genetic regulation of NSC (neural stem cell) dynamics. The role of different pathways in maintaining NSC proliferation, differentiation, and self-renewal are shown. In NSCs, Wnt, MAPK, and Hedgehog signaling acts as a positive regulator of proliferation where its upregulation increases NSC number. TGF-β, Notch, and Hippo pathways act to maintain the quiescent state of NSCs and promote stemness. JAK/STAT pathway is involved in decisions of lineage specification where upregulated signal induces astrocyte differentiation and downregulated signal induces neural differentiation. MAPK is also involved in NSC migration towards the site of injury.

NSC: Wnt signaling also plays a vital role in regulating neural stem cells (NSCs) (Figure 2). The modulation of Wnt signaling causes disruptions in NSC homeostasis and has been associated with pathogenesis involved in Alzheimer’s disease, Parkinson’s syndrome, spinal cord injuries, etc. [11]. Enhancing Wnt signaling expands the neural progenitor pool. Initial studies in a gain of function mice having truncated β-catenin have shown that Wnt drives the proliferation of neural progenitors at the expense of other cell types. Elevated expression of Wnt causes increased neurogenesis owing to the increased proliferation of neuroblasts. Conversely, blocking this cascade causes decreased neurogenesis and an increased number of cells from the glial phenotype [1,12]. Low dose radiation-induced increase in Wnt ligands and β-catenin increases proliferation and differentiation of NSC, along with improved cell survival and reduced apoptosis [13]. 

Adult neurogenesis occurs in two different niches viz subventricular zone (SVZ) and subgranular zone (SGZ). Wnt signaling plays a major role in regulating the proliferation of neural progenitors in the SVZ. Using the mice model, Adachi et al. have shown that increased β-catenin increases the proliferation of Mash1+ cells (marking type C and a subset of type B cells in SVZ). Pharmacological activation of β-catenin also increased the number of newly formed neurons in the olfactory bulb due to the increased number of neural progenitors in the SVZ [14]. Later studies in adult mice SVZ have also produced similar results, where Bowman et al. has shown the presence of Wnt responsive long-term NSCs in the SVZ. They suggest that almost all adult NSC in the SVZ are Wnt responsive in contrast to embryonic ones and can give rise to all major neuronal subtypes in the olfactory bulb. They also report a rare sub-population of Wnt-responsive stem cells in the SGZ of hippocampal dentate gyrus that gave rise to differentiated progenies [15]. Wnt signaling is known to play a role in the symmetric divisions of NSC. It was shown in BAT-gal transgenic reporter mice that Wnt signaling is upregulated during symmetric divisions in response to stroke or during In Vivo regeneration but is absent during asymmetric divisions. They also report that NSC expansion is inhibited upon blocking Wnt, and thus Wnt signaling plays an essential role in maintaining the stem cell pool by controlling symmetric cell divisions [16]. Wnt signaling also plays a major role in lineage specification and differentiation. It is reported that a single adult NSC gives rise to either oligodendroglia or neurons, indicating that they are distinct, separate lineages, and activating canonical Wnt signaling in NSC directs NSC towards oligodendroglia lineage. This Wnt activation does not affect the lineage choice or proliferation among neurogenic clones. These results are recapitulated In Vivo, as well [17]. BDNF (brain-derived neurotrophic factor) is a great contributor to NSC regulation, and it was found that BDNF works through activation of the Wnt/β-catenin pathway. BDNF enhances NSC proliferation, differentiation and cell commitment to neuronal and oligodendrocytic fates. Blocking the Wnt pathway using inhibitors seems to inhibit these observed BDNF phenotypes [18]. 

Other In Vivo evidence for the role of Wnt in NSC comes from Zebrafish. Transcriptome analysis of Zebrafish embryonic NSCs to study glycine-mediated differentiation has identified major signaling pathways involved in NSC differentiation, one of which was Wnt signaling along with others like TGF-β, Shh (Sonic Hedgehog) and Calcium signaling [19]. A study to investigate the role of Wnt on NSC proliferation in the optic tectum of zebrafish also indicated that Wnt signaling suppresses differentiation and enhances proliferation [20]. Another comprehensive study to investigate the role of all 21 Wnt genes in zebrafish neurogenesis was conducted. Their result indicates the expression of multiple Wnt genes in different progenitor zones at different stages of development. The data are indicative of gene redundancy and post-embryonic neurogenesis being regulated by various Wnts at the same location [21]. 

Another model system in which the role of Wnt in NSC has been reported is the planarian system. Planarian LEF/TCF (lymphoid enhancer-binding factor/T-cell specific transcription factor) type transcription factor *Smed-tcf1* has been reported to be primarily expressed in the brain and some stem and progenitor cells that may define the dorsal-lateral neuronal subtypes. Smed-tcf1 RNAi showed regeneration defects in forming proper dorsal-lateral neuronal subtypes, specifically GABAergic neurons. They also exhibited defective negative phototactic behavior and thinner and narrower brain lobes along the medial-lateral axis [22]. Though neural stem cell lineage is not very well defined in the planarians, several studies have demonstrated various signaling pathways affecting the formation of neuronal structures. Various Wnt-family protein encoding genes display regionalized expression along the anterior-posterior (A/P) axis in planarians [23]. They are mainly expressed by planarian muscle cells and helps to determine the A/P axis [24]. Wounding elicits the expression of the Wnt inhibitor notum at anterior-facing wounds. Knockdown of notum in whole animals inhibits the formation of head structures, including cephalic ganglia and photoreceptors in the anterior side [25]. The role of β-catenin and APC has been investigated thoroughly in planarians. Silencing these genes in whole animals produces interesting phenotypes, causing dramatic alterations in the anterior–posterior identity of regenerating tissues. Knockdown of β-catenin results in the formation of head structures on the posterior side of the imputed tissue, resulting in the formation of two-headed animals. On the other hand, APC RNAi animals failed to produce differentiated head structure and regenerate tails from both amputation planes. These animals were devoid of discernible brain tissue at the anterior side and instead exhibited the posterior structures [26].

**Figure 3:**
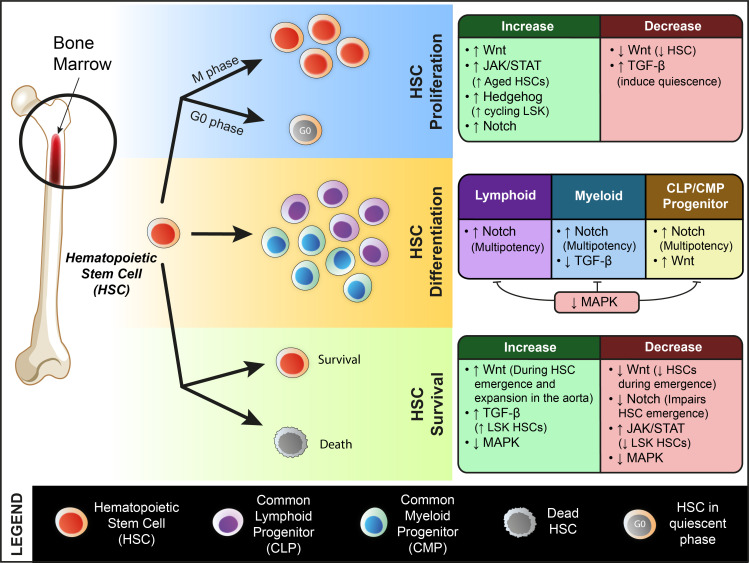
Genetic regulation of HSC (hematopoietic stem cell) dynamics. Hematopoietic stem cells give rise to all the differentiated cell types in the blood. Key signaling pathways regulating the proliferation, differentiation, and self-renewal of HSCs are shown. Upregulation of Wnt signaling generally increases HSC and progenitor proliferation as well as increasing HSC survival and number during embryonic development. Notch signaling maintains the multipotency of the lymphoid and myeloid progenitors. Hedgehog and TGF-β increase LSK HSCs. Increased JAK/STAT, on the other hand, decreases the number of LSK HSCs, decreases repopulation capacity, and increases proliferation. Downregulation of the MAPK pathway causes HSC attrition, with bone marrow aplasia, anemia, leukopenia, and inhibited differentiation. Inhibition of MAPK signaling is reported to promote self-renewal as well.

HSC: The role of Wnt signaling has been extensively studied in hematopoietic development (Figure 3). Reports have shown Wnt signaling’s function in different stages of HSC development, from HSC emergence to fate specification in adults [27]. In Vivo studies in mice have shown that Wnt pathways are active in LT-HSC (long term HSC), which are the more quiescent cells. Studies have reported that high levels of Wnt signaling lead to increased differentiation and loss of stemness. Studies in mice with hypomorphic mutant APC alleles show that HSCs with APC mutations lead to high Wnt levels, which causes increased differentiation and reduced proliferation without any effect on apoptosis. This increase in differentiation without an increase in proliferation effectively leads to loss of stemness. Non-canonical Wnt receptor Ryk also plays a role in HSC self-renewal as studies in Ryk1 loss of function mice models have shown that HSC repopulation is affected, likely due to diminished quiescence and proliferation-induced apoptosis in these cells [28,29]. Wnt signaling is also reported to affect fate specification in progenitors as well. For instance, constitutively active β-catenin in lymphoid and myeloid progenitors causes their increased proliferation and decreases lineage restriction and more stemness [1,30]. On the other hand, there are also studies suggesting that Wnt signaling is dispensable for HSC maintenance [31].

Wnt signaling is required for HSC maintenance in Zebrafish as well [27,32]. No reports indicate any role of canonical Wnt signaling in HSC specification during development. However, non-canonical Wnt signaling in somites has been found to be necessary for the establishment of definitive HSC. This function was found to be modulated through modulating levels of Notch ligands in somites [33]. Wnt has also been shown to be required for HSC emergence and HSC expansion in the aorta [27]. Loss of Wnt9a and overexpression of Wnt antagonist Dkk1 decreases the number of HSCs during the time of HSC emergence. Conversely, overexpression of Wnt8 was found to cause an increase in HSC number during the emergence window [27,34]. 

**Figure 4:**
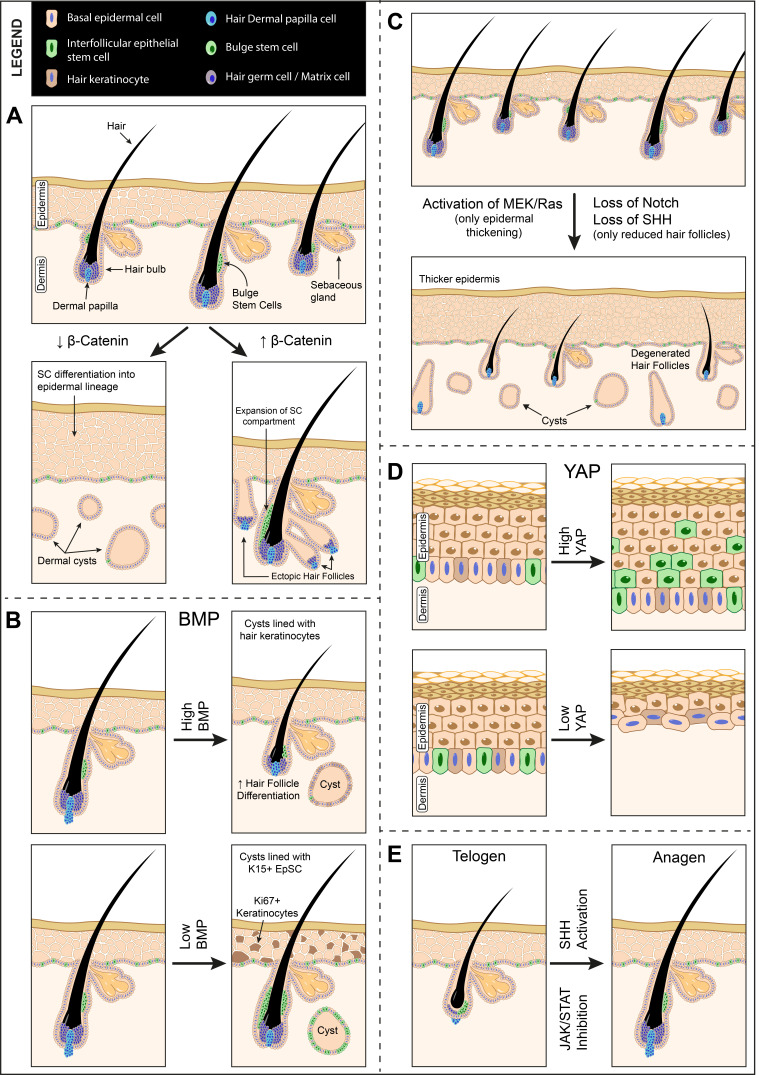
Genetic regulation of EpSc (epidermal stem cell) dynamics. Proliferation, differentiation, and self-renewal in the mammalian epidermis occur mainly due to interfollicular epithelial stem cells and hair bulge stem cells. Signaling pathways regulating these stem cells are indicated. (**A**) represents the effect of the Wnt signaling pathway on EpSc. Active β-catenin expands the stem cell compartment and induces differentiation towards hair follicle lineage. In contrast, loss of β-catenin leads to a complete lack of hair follicles with the formation of dermal cysts and differentiation towards epidermal lineage. (**B**) represents the role of TGF-β signaling. Overactivation of BMP leads to premature differentiation with the formation of cysts having cells of hair follicle lineage, whereas lack of BMP leads to a dramatic increase in stem cells, forming dermal cysts lined with stem cells and hyperplasia of the interfollicular epidermis. (**C**) shows the role of MEK/ERK and Notch signaling. Activation of MEK or Ras leads to hyperproliferation in the epidermis, leading to epidermal thickening. MEK and Notch pathways promote proliferation and inhibit differentiation. Loss of Notch leads to cyst formation and complete lack of hair, along with hair follicle degeneration. Inhibition of Notch leads to premature entry into catagen. (**D**) shows the role of the Hippo pathway. Loss of Hippo or activation of YAP expands the stem cell compartment, decreases differentiation, and leads to multilayered epithelium with abundant progenitor cells. Activation of the Hippo leads to a thinner epidermis formation, with impaired epithelial barrier function and loss of stem cells. (**E**) Shh activation induces anagen phase, whereas loss of Shh leads to reduced hair follicles (**C**). JAK/STAT inhibition also shows accelerated entry into the anagen phase along with increased proliferation in the hair germ area.

EpSC: Wnt signaling is also widely studied in epithelial tissue (Figure 4). One of the earliest In Vivo studies reporting the role of Wnt in skin development utilized the Cre/Lox system to introduce conditional mutations in the epidermis and hair follicles. They note that mutated β -catenin during embryogenesis blocks the formation of placodes that generate hair follicles, resulting in perturbed hair follicle morphogenesis and hairless patches owing to the complete lack of hair follicles in the area. They also report that β-catenin is also required for stem cell differentiation. β-catenin loss disturbs the stem cell compartment in hair follicle bulge and forms dermal cysts. The absence of β-catenin causes stem cells in the cysts to differentiate into epidermal keratinocytes rather than hair keratinocytes [35]. Conversely, activation of β-catenin expands the stem cell compartment and directs differentiation towards hair follicle lineage and leads to ectopic hair follicle formation [36] (Figure 4A). Constitutive activation of β-catenin leads to uncontrolled placode formation, which does not develop into mature hair follicles or show stem cell markers. Some reports that β-catenin activation expands the stem cell population [37]. A regulatory network between Wnt signaling and BMP (bone morphogenetic protein) signaling in bulge stem cells controls the stem cell activity. Upregulated Wnt ligands and reduced BMP signals steer the cell toward hair germ fate and vice versa [38]. Axin 2 expressing basal epidermal stem cells require canonical Wnt signals to proliferate. These cells act in an autocrine manner and produce Wnt ligands by themselves. These cells also produce Wnt inhibitors like Dkk, whose differential diffusion restricts autocrine Wnt activation only to the basal layers [39]. Loss of Wnt signaling by deletion or inhibitors like Dkk lead to premature differentiation and reduced proliferation [40]. Androgen receptor is also reported to negatively regulate β-catenin transcriptional activity in EpScs. [41]. In planarians, Wnt signaling has shown to be involved in promoting the migration of various stem cells, including epidermal lineage-committed stem cells. The epidermal lineage-committed stem cells in planarians are known as zeta-neoblasts. Following the knockdown of notum, a significant reduction in anterior migration of the zeta-neoblasts and their progenies was observed in intact planarians [42], suggesting that notum might be contributing to providing a directional signal to those stem cells and stem cell progenies.

### 2.2. Notch Signaling

Another major pathway involved in stem cell regulation is the Delta/Notch pathway. Notch signaling regulates proliferation, fate determination, cell death etc., in animals. Mammals possess four different single trans-membrane Notch receptors, namely Notch1-4. The ligands (Delta and Serrate/Jagged) for Notch are trans-membrane proteins, and thus Notch signaling acts to transduce a short-range paracrine signal. Ligand binding induces cleavage of Notch Intracellular Domain (NCID) which translocates to the nucleus where they regulate transcriptional complexes mainly containing transcription factor CSL (CBF1, Suppressor of Hairless, Lag-1). 

ISC: Notch signaling is essential to maintain tissue homeostasis and self-renewal of the intestine (Figure 1). Inhibition or disruption of Notch induces cell cycle arrest and differentiation of crypt progenitors into secretory cells. Conversely, overexpression of Notch increases proliferation and impairs differentiation [43,44]. Inhibition of Notch signaling is thought to increase differentiation by attenuating Wnt signaling, which causes secretory cell differentiation in the intestine [45]. Silvia Fre et al. developed a specific CRE-based transgenic mice line to map the lineages which expresses different Notch paralogues. They showed that Notch 1 and 2 are expressed explicitly in crypt stem cells and progenitors, whereas terminally differentiated cells and cells primed towards a secretory fate did not show any Notch activity [46]. Loss of Notch 1 and 2 causes secretory cell hyperplasia and decreased epithelial proliferation. Notch 1 was found to play a primary role in intestinal homeostasis, and stem cell maintenance and Notch 2 plays a major role in crypt regeneration after injury [47]. A recent report shows that Notch inhibition in adult mice impairs ISC function without loss in number and induces apoptosis in the Notch ligand expressing Paneth cells [48]. This effect is followed by a regenerative response and increased Notch activity, replacing the lost Paneth and secretory cells. 

Notch pathway regulates *Drosophila* midgut development from larval to pupal and adult stages. The initial characterization in *Drosophila* showed that Notch signaling is essential for the differentiation of ISCs into enterocytes or enteroendocrine cells of the intestine. It was observed that the fate specification happens based on the levels of vesicular Delta. ISC having high Delta activates Notch which in turn downregulates Delta in the daughter cells that become enterocytes. ISCs have low levels of cytoplasmic vesicular Delta, which specifies the daughter cells to differentiate into enteroendocrine cells. Notch was also found to be necessary for the fate specifications of ISC daughter cells as well. It was also observed that a reduction in Notch leads to an increase in the midgut progenitors, whereas its activation leads to a decrease in proliferation [49,50,51]. 

NSC: Notch signaling plays an essential role in neural stem cells as well (Figure 2). The proper maintenance, inhibition of premature differentiation, and self-renewal of NSC are primarily dependent on Notch signals. Notch signaling has been reported to be highly active in type B cells and type 1 cells of the SVZ and SGZ, respectively [52]. In studies using Nestin-Cre/Fbxw7F/F mice, which is mutant for Fbxw7f (degrades Notch family), the accumulation of Notch causes morphological changes in brain structure, increases the NSC numbers in the ventricular zone, increases stemness, and induces differentiation orientation towards Astrocytes. Notch was found to be necessary to maintain the proper ratio between neurons and astrocytes [53,54]. Similarly, in a study using conditional knock-out mice that lack RBPJ (J kappa-recombination signal-binding protein)/CSL, an intracellular signal mediator of all the Notch receptors, the neural stem/progenitor cells in telencephalon were depleted owing to premature differentiation of type B cells into neurons [55]. The authors report a transient burst in NSC proliferation in the SVZ following RBPJ deletion, increased neurogenesis, and finally depletion of NSCs. Similar results were observed upon depletion of RBPJκ in NSCs in the hippocampal SGZ. Inactivation of RBPJκ resulted in increased neuronal differentiation, depletion of Sox2+ stem cell pool, and reduced self-renewal [56]. Curcumin-induced upregulation of Notch1 and Hes1 also increases the number of NSC and newly formed neurons in the hippocampal region [57]. EGFR-dependent regulation of Notch signaling in the SVZ is also reported to increase the NG2+ progenitor cell number along with the reduction in GFAP+ NSC number and self-renewal [58]. VEGF-induced upregulation of Notch is also reported to cause a significant increase in proliferation and associated neurogenesis [59].

Studies using Zebrafish report that the levels of Notch activity determine the shift between quiescence and dividing states in neural progenitors. Quiescence is promoted by inducing Notch, whereas inhibition of Notch signals causes progenitors to re-enter the dividing state and drives them towards neuronal commitment [60,61]. These results have been recapitulated in adult mice SGZ as well [61]. A study in *Drosophila* has shown that Notch signaling is vital in controlling apoptosis of neural progenitors. AbdA (Abdominal A) homeobox protein, which provides special cues for cell death, is found to be regulated by Notch. This Notch signaling was activated by Delta present on the neighboring stem cell progenies [62]. Furthermore, Notch regulated the transit between symmetric proliferative division and asymmetric differentiative division in neuroepithelial cells of *Drosophila*. Inhibition of Notch in these cells causes a shift from symmetric to asymmetric division [63]. 

HSC: Earlier studies have shown that Notch is active in HSCs and as the cell differentiates, the levels of Notch decreases. Notch signaling is also required for the HSC emergence from hemogenic endothelium during embryonic development [64]. Induction of Notch was found to provide multipotency in progenitors in vitro [1] (Figure 3). Induction of Notch signaling In Vivo by overexpressing Jagged1 in osteoblasts in mice causes an increase in the number of HSCs in bone marrow [65]. Notch is also required for fate decision between B vs T-cell lineages [64,66,67]. HSC crosstalk with niche cells, including endothelial cells, is found to be mediated by Notch signals. Bone marrow endothelial cells express Notch and Jagged, and their capacity to maintain HSCs is maintained through Notch signals. Deleting Jagged in endothelial cells decreases the number of functional HSCs due to reduced Notch signaling in those HSCs [64]. Other than endothelial cells, Mesenchymal stromal cells, osteoblast cells, etc., were also found to regulate HSC dynamics through Notch signaling [68]. The Notch pathway concerning HSCs has been well explored in *Drosophila* and Zebrafish. Notch is necessary for early HSC specification in Zebrafish embryos [69]. Notch activation of hemogenic endothelium by primary cilia in endothelial cells specifies proper HSCs and progenitor cells during embryogenesis [70]. Similar results are observed during endothelial to hematopoietic transition (EHT) in Zebrafish, when evi1, a Zebrafish transcriptional regulator, is suppressed. Suppression of Evi1 in aortic endothelial cells impairs HSC emergence and EHT. Evi1 functions through activation of Notch [71]. EHT is also affected by the Hypoxia-inducible factor HIF1α and HIF2α, which modulates downstream Notch signaling. Overexpression of Notch rescues the mutant HIF phenotypes during EHT [72].

EpSC: Notch pathway components are also widely expressed in epidermal stem cells and this pathway mainly regulates differentiation (Figure 4). Early reports show that Notch signaling is essential for postnatal maintenance of hair follicles and sebaceous glands. Deletion of Notch components during embryonic development did not have any significant effects. Activation of this pathway regulates terminal differentiation of intrafollicular epidermis into hair follicle lineages [73]. In Notch1 null mice, the first hair cycle is characterized by a short anagen phase (the growth phase of hair cycle) followed by a premature entry into catagen phase (short transitional phase before the resting phase) [74]. Inactive Notch in adult mice results in complete loss of hair and cyst formation (Figure 4C). Double and triple deletions of Notch1, 2 and 3 show increasing and strong phenotypes observed in Notch1 null phenotypes [75]. Overexpression of Notch 1 in suprabasal epidermal keratinocytes and IRS (inner root sheath) leads to hyperplasia of differentiated cells in the epidermis, abnormal differentiation of IRS, and hair formation [76]. This hyperplasia is not associated with an increase in the proliferative compartment but an increase in differentiated cells. These studies seem to indicate that activation and deletion of Notch signaling lead to hyperplasia phenotypes. The Notch pathway is also known to interact with Wnt and Vit A pathways to regulate fate decisions. Furthermore, knockdown of JAG1 (Jagged 1) causes reduced proliferation and defective differentiation in basal layer cells. Lower expression of HES1 (hairy and enhancer of split 1, a Notch target gene) reduces proliferation of skin EpSC [77]. 

A recent study using the rabbit ear scar model reports that basic FGF (fibroblast growth factor) reduces scar formation by inhibiting EpSC differentiation towards myofibroblast lineage. This inhibition of differentiation was brought about by the activation of Notch/Jagged1 [78]. This same group has reported earlier that Notch ligand Jagged1 promotes EpSC proliferation and decreases differentiation through the HES1 gene, using a diabetic mice model for wound healing. The in vitro data also suggest that overexpression of JAG1 promotes EpSC migration and its knockdown significantly reduces the migratory ability of these cells [79]. 

### 2.3. JAK/STAT Pathway

The Janus Kinase (JAK)/Signal transducers and activators of transcription (STAT) pathway is one of the conserved pleiotropic signaling cascades which transduces a variety of signals including cytokine signaling and growth factor signaling involved in development and stem cell regulation, normal tissue homeostasis, growth, immunity etc. Janus Kinases are the cell surface receptors which, upon activation, transduce the signal through STAT, which is activated, dimerized, and translocated to the nucleus for further transcriptional control. 

ISC: In a study using *Drosophila* strains to explore JAK/STAT signaling, it was observed that JAK/STAT pathway is normally active in ISCs and their immediate progenitors, but inactive in terminally differentiated cells. Researchers have also shown that lack of this signaling in individual ISC lineages results in failure of cell fate specifications. JAK/STAT signaling is an important regulator of proliferation, but not in self-renewal [80] (Figure 1). Reports show that enterocytes, when subjected to apoptosis, infection or stress, produce cytokines called Unpaireds (UPD) which activate JAK/STAT signaling in ISC, causing rapid proliferation. This pathway also induces progenitor cell differentiation, partly through Delta/Notch signaling activation as well, thus replacing damaged tissue [81,82]. There are also reports showing the JAK/STAT and Wg pathways acting parallelly to regulate ISC proliferation. The JAK/STAT pathway components like Dome, Hop and STAT92E are also cell-autonomously required for ISC maintenance and proliferation. The role of JAK/STAT signaling is more important in proper differentiation of daughter cells rather than ISC divisions in healthy flies. Hop and Dome mutant ISC undergoes defective differentiation and the daughter cells are blocked at the enteroblast stage, which is a progenitor that give rise to enterocytes and enteroendocrine cells [83,84]. 

A very recent report suggests the role of JAK/STAT signaling in epigenetic modifications. Deletion of Histone deacetylases HDAC1 and HDAC2 are causes of proliferative and differentiation defects in the murine intestinal epithelium. Using enteroid cultures derived from villin-Cre dual Hdac1^−/−^ Hdac2^−/−^ mice, authors have shown that JAK/STAT inhibition in mutants partially restores the intestinal epithelium, with an increase in Paneth cells, better barrier and polarity as opposed to the control mutants with decreased Paneth and goblet cells, impaired polarity, and impaired proliferation [85].

NSC: Modulation of JAK/STAT influences the proliferation and differentiation of neuronal stem and progenitors (Figure 2). They play a direct role in neurogenesis and scar formation during injury. They are reported to be activated during CNS (Central Nervous System) injury. Their activation also induces astrocyte differentiation in NSCs and progenitors. There are reports indicating that Prolactin and gp130 (IL6 family of ligand) allow differentiation of astrocytes through JAK/STAT pathway [86]. 

In *Drosophila*, JAK/STAT controls the balance between self-renewal and differentiation of NSCs. JAK/STAT signaling prevents the development of Neuroepithelial (NE) stem cells to neuroblasts (NB) during late fly development. Loss of this signaling causes differentiation in NE stem cells and leads to NE and NB depletion [87,88,89,90]. Similar results are observed in the mammalian system as well. A recent study focused on JAK/STAT signaling during ethanol-induced neurodegeneration found that inhibition of JAK and STAT3 together significantly increased the differentiation rate of NSC, without any effect on proliferation. However, these roles were found to be reversed under neurodegeneration conditions. Inhibition of the signaling accelerated the cell cycle and increased the number of neural progenitors [91]. In another report studying the role of DNA damage in NSC homeostasis, it was observed that NSCs enter cellular senescence characterized by lack of proliferation, loss of stem cell markers and astrocyte differentiation. This was found to be mediated by a non-canonical BMP signaling acting through JAK/STAT pathway [92]. There are also reports indicating that downregulation of JAK/STAT brings about inhibition of astrocyte differentiation [93] and facilitates neuronal differentiation [94].

HSC: During *Drosophila* definitive hematopoiesis in the lymph glands, JAK/STAT signaling is required for the maintenance of prohemocytes in its undifferentiated state. JAK/STAT signals maintain them in a primitive self-renewing state and differentiation is induced on inactivation of this signaling. JAK/STAT is also required for proliferation and production of lamellocytes, a specific type of blood cell other than the common hemocytes and crystal cells [95]. Decline in stem cell function associated with aging has also been shown to be influenced by JAK/STAT signaling in mice. Constitutive activation of JAK3 induced prolonged proliferation and a functionally impaired subpopulation of aged HSCs, characterized by p53 upregulation [96]. The role of JAKs in maintaining HSC function has been reported earlier as well. In studies using a conditional knock-in mice model, it was observed that JAK2^V617F^ mice (gain of function mutation associated with neoplasm) show a reduction in the numbers of lineage^−^ Sca-1^+^c-Kit^+^ (LSK) cells (Figure 3). They were also found to be associated with reduced cell cycling, increased DNA damage, and reduced DNA damage-induced apoptosis. This mutant also showed impaired LT-HSC function and significantly reduced repopulating capacity during competitive repopulation assays [97]. The overexpression of this mutant in megakaryocyte primed progenitors was found to be sufficient to expand hematopoietic cells and induce myeloproliferative neoplasms [98]. In Zebrafish, JAK2a was found to initiate primitive hematopoiesis and is involved in cell fate decisions of early progenitors. Activation of it in zebrafish embryos resulted in a significant increase in hematopoiesis marked by the increase in gene expression of hematopoiesis related genes [99].

EpSC: One of the earliest In Vivo studies to investigate the role of JAK/STAT signaling in mice utilized a Cre Lox system to knock out STAT3 in keratinocytes. The report indicates that these mutants showed severe defects in skin remodeling after wound healing and impaired hair cycle. STAT3 disruption does not show any developmental skin defects, however, the second anagen phase was severely impaired with skin fibrosis and inflammatory cell infiltration. Wound healing was severely impaired in these mice with defects in re-epithelialization. This defect was due to defects in epidermal regeneration since granulation, inflammation, and neovascularization after wounding were found to be normal [100]. JAK/STAT signaling regulates the entry of hair follicle populations into the anagen and telogen phases of hair growth (Figure 4). It is reported that inhibition of JAK/STAT pathway using small molecule inhibitors in mice and human skin induces entry into anagen leading to hair growth. This was accompanied by a significant proliferation in the hair germ area, suggesting proliferation of progenitor stem cell populations [101] (Figure 4E). Quiescence of hair follicle stem cells via prolactin during pregnancy/lactation is also found to be mediated by JAK/STAT5 signaling [102]. 

### 2.4. Hippo Pathway

Hippo signaling is a highly conserved cascade generally involved in the control of organ size, cell proliferation, apoptosis, stem cell self-renewal, and cancer. Hippo (MST1/2 in mammals) is a protein kinase that phosphorylates and activates Warts (LATS1/2 in mammals), which in turn inactivates transcriptional co-activators YAP (Yes-associated protein) and TAZ (transcriptional coactivator with PDZ-binding motif) by inhibitory phosphorylation. The hippo pathway is generally considered a tumor-suppressor pathway since mutations in this pathway result in an over-proliferation phenotype. 

ISC: The Hippo YAP regulation of intestinal epithelia is complex and depends on many additional factors. Studies in *Drosophila* have established the role of Hippo signaling in regulating organ size and stem cell dynamics. Hippo signaling was initially identified as one of the signaling causing tumorigenesis in *Drosophila* intestines. The loss of Hippo signaling in precursor cells or epithelial cells stimulates ISC proliferation, suggesting Hippo signaling in adult cells is necessary to control ISC proliferation (Figure 1). Furthermore, loss of the Hippo causes ectopic expression of multiple ligands of the EGFR (epidermal growth factor receptor) and JAK/STAT pathways which drives ISC proliferation [10,103]. In mice models, YAP’s transgenic expression induces proliferation of stem cell compartments, dysplasia along the entire intestinal epithelium, and increased proliferation of crypt and villus marked by an increase in PCNA (proliferating cell nuclear antigen) protein levels. Activation of YAP1 in these mice also led to an absence of differentiated enterocytes, goblet cells, and Paneth cells. This lack of differentiated cell types was due to an expansion of Hes1+ undifferentiated crypt progenitor cells, whose expansion gradually replaced all the differentiated cells. This effect was found to be reversible as the inactivation of YAP1 brings back all the differentiated cell types [104,105]. 

Studies in mice report that ablation of MST1/2 from intestinal epithelium causes expansion of stem cell compartment and loss of secretory cells. They also report enhanced nuclear localization of YAP along with activation of β-catenin and Notch signaling [106]. Here, the ablation of the YAP allele significantly suppressed the proliferation phenotype of MST1/2 knockout animals. YAP/TAZ gene transfer into mice intestinal cells has shown that these Hippo effectors induce proliferation of stem/progenitor cells and differentiation into goblet cells [107]. Cai et al. has reported YAP protein overexpression during intestinal regeneration after injury, without much change in Hippo signaling activity. They report that YAP is dispensable for normal intestinal development and homeostasis, but they are essential for regeneration after injury [108]. Hippo signaling is suggested to keep the YAP protein phosphorylated and inactive under normal conditions, and deregulation of Hippo causes YAP activation, which induces a hyperproliferative state. However, a later report showed a growth-suppressive function for YAP and that its depletion causes the proliferative phenotype following damage. This phenotype was found to be associated with YAP mediated restriction of Wnt signaling [109]. These results suggest the indispensable role of Hippo signaling in intestinal homeostasis while indicating a contextual role of YAP protein.

NSC: Hippo signaling restricts *Drosophila* neuroblast proliferation and thus regulates brain size. It also controls the cell cycle time, ratio of different neural cell types formed, reactivation from quiescence, and differentiation. Hippo signaling maintains neuroblasts in a quiescent state (Figure 2). Hippo or Warts (the protein kinase phosphorylated by Hippo) deficient neuroblasts undergo premature exit from quiescence and enter the cell cycle [110,111,112,113]. Loss of Hippo or Warts in *Drosophila* initiates premature proliferation in NSCs. It is mediated by an increased nuclear localization of the downstream effector Yorkie, which is absent in the quiescent NSC nucleus. Yorkie is necessary to cause proliferation in NSCs as Yorkie mutants fail to show any NSC reactivation from quiescence. These mutants do not show any mitotic cells or cell growth, whereas a constitutively active form of Yorkie is sufficient to reactivate and induce proliferation in NSC [111]. 

In Vivo evidence from Chick neural tubes, *Xenopus*, and Zebrafish indicate a co-localization of YAP with SOX2+ cells and expansion of SOX2+ progenitors and PAX3+ neural crest progenitors associated with YAP overexpression. YAP was also found to repress genes involved in differentiation [114,115]. The expansion in the number of neural stem/progenitor cells is caused by increased proliferation and decreased differentiation. Recent transcriptome analysis in mice shows that YAP/TAZ activation by LATS1/2 deletion induces hyper transcription and upregulated genes involved in the proliferation of neural progenitors. However, this proliferative phenotype was found to be transient and was followed by massive apoptosis of neural progenitors with replicative stress and DNA damage [116]. A very recent report showed that YAP overexpression in neural stem cells enhances self-renewal and proliferation and increases FGFR receptor expression, which upregulates MAPK and AKT signaling pathways [117].

HSC: The role of Hippo signaling in HSCs is less explored. Reports indicate that Hippo signaling might not play any significant role in the Hematopoietic system, although Hippo plays a very prominent role in other adult stem cell and organ systems. Studies in a transgenic mice model with inducible YAP1 exclusively in the Hematopoietic tissue show that induced expression of wild type or constitutively active YAP1 does not influence HSC dynamics or function In Vivo. No changes were observed in the lineage distribution, progenitor cell compartment or function in terms of transplantation efficiency [118]. This study concentrated on the role of YAP1. Other downstream signal components might still play an important role in HSC dynamics as mRNA levels indicate the presence of Hippo pathway components in HSC lineages. 

EpSC: The Hippo pathway’s downstream effector YAP is known to regulate proliferation and tissue expansion in epidermal stem cells. YAP is localized in the proliferative basal cells of hair follicular bulge and interfollicular epidermis, although nuclear localization reduces postnatally [119]. Using loss and gain of function mice models, Schlegelmilch et al. showed that YAP activation (via enhanced nuclear localization of YAP1) expands the EpSC compartment (Figure 4D). These mutant mice develop multilayered epithelium with an abundance in progenitor cell populations. These mice did not show an expansion in hair follicular/bulge stem cells but only in interfollicular epidermal stem/progenitor cells [120]. Loss of YAP causes developmental defects with thinner and fragile skin, loss of epidermal barrier function (increased permeability) and a decrease in the number of proliferating basal cells (Figure 4D). However, these phenotypes are not recapitulated upon deletion of MST1/2, which acts upstream of YAP. This indicates the regulation of YAP by a pathway other than the canonical Hippo pathway. Further studies found that YAP was regulated by its interaction with α-catenin [120]. Other reports also indicate that YAP overexpression causes an expansion of the proliferative compartment, decreased apoptosis, and reduced differentiation [119,121]. Inactivation of SAV1 (upstream regulator of Hippo pathway) also produces a hyperproliferative phenotype similar to YAP’s nuclear localization, with progenitor cell expansion, reduced differentiation, and decreased apoptosis [122]. The hippo pathway components interact with other signaling pathway components like Hedgehog, Wnt, and Notch to regulate epidermal stem cells. Activation of Hedgehog signaling (GLI2) increases the YAP levels in the nucleus of Hedgehog activated epidermis, indicating a positive regulation of Hippo signaling by Hedgehog signaling. Conversely, activation of YAP in the epidermis leads to increased levels of GLI2. These increased levels of YAP also show similar phenotypes, as explained earlier [123]. Another in vitro report suggests that epidermal stemness is promoted by activated YAP/TAZ, which acts by inhibiting Notch signaling, which is a major player in the differentiation program [124]. YAP also interacts with the Wnt pathway by inducing Wnt16 production to drive keratinocyte proliferation in the epidermis [125].

The hippo pathway also influences epidermal lineage cells in planarians. De Sousa et al. reported that hippo knockdown by RNAi impairs the differentiation of epidermal lineage cells. Loss of hippo causes co-expression of markers of different epidermal lineages and causes mis-localization of these cell types. Hippo RNAi produced unpigmented overgrowth regions containing many mitotic cells in the animal’s periphery. In planarians, peripheral areas are formed of post-mitotic progenitor cells. Hippo RNAi also impairs the fate maintenance indicated by the presence of differentiated epidermal markers (vim+) in the mesenchyme, where usually undifferentiated progenies are observed. These animals also show an increase in the ζ neoblast (epidermal precursor) marker expression. Hippo knockdown is also associated with decreased apoptosis, cell cycle arrest, and dedifferentiation of post-mitotic cells [126]. 

### 2.5. TGF-β Super-Family

TGF-β family signaling is a major player in the regulation of cell growth, differentiation, and development. The TGF-β superfamily of molecules can be divided into the BMP group and TGF-β group. The binding of a suitable ligand initiates signaling that induces oligomerization of Ser/Thr kinase receptor. This leads to phosphorylation of SMAD2 and 3 (for TGF-β ligands) and SMAD1,5,9 (for BMP pathway). Phosphorylated SMAD proteins are associated with the common SMAD4 and are translocated to the nucleus, where they partner with various transcription factors and regulate gene expression. 

ISC: The TGF-β signaling pathways play a crucial role in ISCs (Figure 1). BMP signaling is generally more potent in the differentiated cells of the intestine. A study using mice in which Bmpr1a (type 1 BMP receptor) can be conditionally inactivated using the interferon-inducible Mx1-cre line shows that the conditional inactivation of this receptor disrupts the intestinal homeostasis along with an expansion of the stem and progenitor populations. They have shown that this receptor inactivation induces ISC self-renewal, with mice having five times normal ISC and eventually causing intestinal polyposis in those mice. Another interesting finding in the paper is that BMP signaling suppresses the Wnt signaling to maintain the balance between self-renewal and differentiation [127]. Another study in mice supports this BMP-associated hyperproliferation of Lrg5+ ISC but reports a Wnt/β-catenin independent inhibition of ISC stemness through a Smad-mediated repression of numerous stem cell-related genes [128]. 

BMP signaling has been studied in the *Drosophila* system as well. It was found that the two BMP ligands, Dpp and Gbb, were increased upon injury in *Drosophila* enterocytes, leading to an elevated BMP signaling in progenitor cells through an autocrine–paracrine mechanism which in turn causes an expansion of self-renewing ISCs. A negative feedback mechanism to maintain the number of ISCs was also observed where the BMP signaling in enterocytes inhibits the Dpp and Gbb production [129,130]. Studies have shown that the ISC response to Dpp is achieved by differential activation of Sax and Tkv receptors and their downstream effectors SMOX and Mad. In ISC, Sax is constitutively expressed and hence presents the initial response to Dpp signaling inducing proliferation of ISCs via SMOX. Tkv receptors, on the other hand, are expressed only during the later phase of the response and signaling via Tkv and Mad diverts the signaling from Sax and induces quiescence in ISCs, bringing the proliferative stage back to normal [131,132].

NSC: In the nervous system, TGF-beta signaling plays a variety of roles (Figure 2). It acts as a neuroprotective and controls NSC proliferation, whereas it acts as a mitogen in the case of a tumor. In the normal brain, TGF-β generally has an anti-proliferative function. TGF-β is known to inhibit the proliferation of astrocytes, microglia, oligodendroglia, and induce its differentiation. In vitro experiments have shown that TGF-β reduces neural stem and progenitor cell proliferation in a dose-dependent manner [133]. Studies in mice have shown that TGF-β signaling negatively regulates the self-renewal of midbrain neuroepithelial stem cells. TGF-β inactivation was found to enhance the proliferation of SOX2-positive neuroepithelium of the mutant dorsal midbrain. It specifically increased the proliferating progenitor cells as no apparent change in apoptosis or differentiation rates was observed. Cells in the mutant midbrain had a shorter cell cycle time, and an increased proportion of cells in the S phase. Thus, TGF-β might be controlling the decision of cell cycle exit where it promotes the exit from the cell cycle rather than continuing to the next cycle of the division [134]. Similar reports have shown that inhibition of TGF-β signaling In Vivo using an antibody against TGF-β or by a small molecule inhibitor significantly increased the number of proliferating neural stem/progenitor cells, improved neurogenesis in aged and irradiated mice with an increase in the number of neuroblasts, and reduced apoptosis [135]. TGF-β also promotes stem cell quiescence [136]. These authors report that TGF-β1 promotes stem cell quiescence and is also responsible for cell survival through their control over proliferation. The authors suggest a unified theory where TGFβ-1 signaling in the NSC niche contributes to quiescence, differentiation, and cell survival. 

In planarians, a recent study has demonstrated that inhibition of Wnt and TGF-β signaling during growth alters the patterning of mechanosensory neurons—a neural subpopulation that is distributed in accordance with worm size and modulates fission behavior [137]. Hence, it is suggested that TGF-β and Wnt has an important role in regulating size-dependent behavior in planarians and there is an interdependence between patterning, growth and neurological function in these animals.

HSC: The role of TGF-β signaling in HSC has been studied extensively over the past few decades. BMP signaling is essential to maintain HSC niche size, and it also affects the reconstitution ability of HSCs. The role of BMP on HSC could also be indirect. BMP is known to influence the number of osteoblastic cells, which constitute the endosteal niche where more primitive LT-HSCs reside [1,138]. Using a temperature-inducible BMP dominant-negative Zebrafish model, it is reported that BMP signaling is required for initiation and maintenance of definitive HSC emergence during development [139]. In vitro studies have shown the role of TGF-β as a negative regulator of HSC growth. It may also induce quiescence in HSCs [140]. On the contrary, certain studies have shown TGFβ-2 to be a positive regulator of LSK number In Vivo [141] (Figure 3). Furthermore, Tgfb2^+/−^ or TGF-β type 2 receptor (Tgfbr2)-deficient mice also display a reduced potential for competitive repopulation [141,142]. TGF-β signaling influences every cell type in the hematopoietic lineage and its effect varies on different cell types. For instance, TGF-β acts as a negative regulator of myelopoiesis as TGF-β^−/−^ mice show an increased number of immature neutrophils, monocytes, and platelets. It is also a necessary pathway for the maintenance of quiescence In Vivo [140]. 

EpSC: BMP/TGF-β signaling plays an essential role in stem cell activation, differentiation, hair follicle morphogenesis etc. (Figure 4). While the Wnt signaling mainly controls the activation and growth phase of the hair cycle, TGF-β/BMP signaling controls the regression and resting phase. BMP signaling in the hair follicle niche has been known to inhibit follicle stem cell activation and induce an anagen phase. Bmpr1a mutant mice hair follicles show a dramatic increase in the number of EpSC (Figure 4B). The expression of Noggin or ablation of BMP signaling by a Bmpr1a mutant in EpSC blocks the BMP signaling, which coincides with stem cell activation and expansion during the anagen phase [143,144,145,146]. This indicates that BMP signaling in the skin acts to restrict the activation and proliferation of EpSC and maintains them in a quiescent state. Overactivation of Bmpr1a also causes premature terminal differentiation [144]. Thus, BMP signaling needs to be active such that it maintains the cells in a quiescent state but does not induce terminal differentiation. The authors suggest a theory where the hair cycle is activated when BMP signals drop below this particular threshold level and when the levels cross above, differentiation programs are activated. Disruption of SMAD4 (common co-activator of TGF-β/BMP signaling) was also found to produce similar phenotypes, with hyperplasia of interfollicular epidermis and activation of follicular stem cells leading to stem cell exhaustion [147]. The paracrine TGF-β signaling, which acts through SMAD2/3 instead of the common SMAD4, acts antagonistically to BMP signaling in hair follicle stem cells. Here, loss of TGF-β2 signaling causes a delay in stem cell activation. Researchers have uncovered that Tmeff1 is a direct target of TGF-β2 signaling, which antagonizes BMP signaling to activate stem cells [148]. Telomere dysfunction impairing EpSC specification and differentiation is suggested to act through disruption of BMP/pSmad/P63 signaling [149].

The zeta-neoblasts (epidermal stem cells) in planarians express various genes depending on their dorsal–ventral position in the animals. The expression of PRDM1-1 is exclusive in dorsal zeta-neoblasts, whereas ventral zeta-neoblasts express Kal-1. It has been demonstrated that bmp4 expression from dorsal muscle cells regulates the polarization of the dorsal–ventral axis in planarians [24]. Knockdown of bmp4 induces Kal-1 expression in dorsal zeta-neoblasts, suggesting that BMP signaling is involved in repressing the expression of ventral epidermal genes in dorsal epidermal lineage cells [150]. Thus, it helps to generate regionally appropriate mature epidermal cells.

### 2.6. Hedgehog Pathway

Hedgehog was initially discovered as a mutant involved in segment polarity in *Drosophila* and was later found to be important in the developmental context. Three types of Hedgehog proteins are present in mammalian cells, viz. Sonic (Shh), Indian (Ihh), and Desert Hedgehog (Dhh). The membrane receptor for hedgehog, called Patched, inhibits the downstream signaling in the absence of the hedgehog ligand. Binding of hedgehog to Patched lifts the inhibitory effect of Patched, leading to activation of transcription factor Gli.

ISC: Studies in mice have shown that hedgehog signaling is essential in intestinal stem cell maintenance. Reports show the Indian Hedgehog (Ihh) expression in mice mature colonocytes, and they regulate colonic epithelial differentiation. Colonic epithelium of mice null for Indian hedgehog display multilayered cells which do not form crypts. They also show an increase in epithelial cell proliferation [151] (Figure 1). Studies using mice with intestinal epithelial-specific disruption of Ihh reported increased Olfm4^+^ ISC proliferation, disrupted mesenchymal architecture, reductions in numbers of crypt myofibroblast, and increased Wnt signaling [152]. Shh signaling also functions to restrict the Wnt targets to the base of crypts. They have also reported deregulation of BMP signaling upon Ihh disruption [151,152]. Other studies in models of mice for Sonic Hedgehog (Shh) have generated similar results with decreased crypt proliferation, decreased ileal crypt/villus length, and reduced number of goblet cells [153,154]. There are also some contradicting results where Ihh disruption leads to a decrease in progenitor cell proliferation. These Shh and Ihh mutant mice showed defective gastrointestinal development, with a gross reduction in size. Shh mutants showed an overgrown epithelium with overgrown villi, whereas Ihh mutants displayed a dilated intestine with a thin wall and smaller villi. Ihh^−/−^ mutants also show a substantial reduction in cell proliferation in the stem cell compartment [155]. Here, the authors did not look for any specific ISC marker for proliferation; instead, they used PCNA as a marker for proliferating cells in the stem cell compartment at the base of the crypts. 

Studies in *Drosophila* also indicate that Hedgehog controls ISC proliferation. However, it is suggested that the pathway might not be necessary under normal homeostasis. Reports indicate that Hedgehog plays a major role during stress induced by damage or ageing [156]. An injury induces the Hedgehog signaling in enteroblast cells, which renders the proliferation of ISCs indirectly through the JAK/STAT pathway [157]. 

Hedgehog transcription factor Gli1 also regulates the differentiation in planarians. RNAi-mediated knockdown of Gli1 in *Schmidtea mediterranea* causes reduced goblet cell differentiation in the developing intestinal regions of amputated head, tail, and trunk fragments. Reduction in goblet cell numbers was also observed upon Gli1 knockdown in uninjured animals. This effect was more profound in uninjured animals in the anterior, posterior, and lateral intestinal branches compared to the primary medial branch. Therefore, Gli transcription factor might be necessary for the normal homeostatic development of lateral intestinal branches. In contrast, it is essential to develop both lateral and medial branches in regenerating animals [158].

NSC: The role of Shh in Neural stem cells came to light after discovering Gli factors in the NSC niche, which are the effectors of Shh signaling. Several studies have identified the role of Shh signaling in maintaining NSC in sub-granular and subventricular zones of the brain. In Vivo experiments have demonstrated that the Shh pathway promotes proliferation, and Shh inhibition blocks proliferation [159,160,161,162] (Figure 2). Recent studies have shown that Shh signaling plays an active role in regulating the proliferation and quiescence of NSCs in ventricular and subventricular zones in the adult brain. Deletion of the Hedgehog receptor was found to upregulate the signaling and accumulation of quiescent NSCs and loss of activated NSC and their progeny [163]. Recent reports from studies in mice show that the Shh pathway also regulates NSC lineage progression during development. The switch in the state of neocortical NSCs between glutamatergic projection neurons and GABAergic interneurons, cortical oligodendrocytes, and astrocytes was brought about by Shh signaling. It was both necessary and sufficient to induce the generation of GSX2+ progenitors (the tripotential population that can give rise to the above-said interneurons, astrocytes, and oligodendrocytes) [164].

HSC: In the hematopoietic system, hedgehog signaling has been implicated from the developmental stage itself. Studies investigating the signaling pathways involved in the induction of the HSC program in dorsal aorta (DA) in Zebrafish found that hedgehog signaling maintains the arterial program characteristic of the DA roof. Hedgehog signaling restricts the HSC formation to the DA ventral wall through transcriptional activation of tbx20. Transcription factor tbx20 and HSC emergence are mutually exclusive. Tbx20 is restricted to the dorsal side, whereas definitive hematopoiesis initiates at the ventral side. Hedgehog activates tbx20 on the dorsal side, and this distance from hedgehog signaling, along with BMP signaling, is thought to contribute towards the ventral localization of HSC [139]. Similar studies were conducted in mice using explant cultures of dorsal and ventral tissue along with aorta gonad mesonephros (AGM) HSC. Ventral tissue was found to increase the AGM HSC activity, whereas dorsal tissue decreases it. This was caused by instructive hedgehog signaling from the tissue [165]. 

Hedgehog signaling regulates the adult HSCs as well. The Hg regulates the HSC cell cycle during homeostatic as well as during acute regenerating conditions (Figure 3). A study in ptc1 ^+/−^ mice reported that Hedgehog activation expands primitive bone marrow LSK HSCs without affecting mature cells. However, this effect was at the expense of HSCs, resulting in an overall decline in the regenerative capacity due to lack of self-renewal and deregulation of cell-cycle related genes. Inhibition of this signaling was found to rescue the regeneration defect, suggesting hedgehog’s specificity in self-renewal In Vivo [166]. There are also reports that hedgehog signaling is dispensable for adult HSC function. Studies using mice for deletion/activation of Smo have found that hedgehog signaling has no significant effect on adult hematopoiesis, including long-term repopulation and cell cycle status of the stem and progenitor cells [167,168]. 

EpSc: Shh signaling is essential for the formation of hair follicles present in hair plaques during development (Figure 4). It is also crucial during the anagen phase of the hair cycle. Blocking Shh hampers the progression of the anagen cycle and hair regeneration. The Shh null mice develop skin with reduced hair follicles (Figure 4C). Conversely, Shh or other agonists accelerates the progression from telogen (resting phase of the hair cycle) to anagen [169,170,171] (Figure 4E). However, these agonists do not cause any change in proliferation and differentiation of the epidermis or the endogenous Hedgehog signaling [171]. SMO and GLI are essential in maintaining stem cell niche and identity in mice [172]. GLI2 mutant mice are reported to show hair follicle development arrest with reduced cell proliferation in the follicular epithelium [173]. Mutations in Patched and Smo are also known to cause basal cell carcinomas (BCC) in humans, and overexpression of Hg signaling in mice also induces BCC following stem cell expansion [174]. Overexpression studies in mice have shown that Ihh overexpression does not cause any noticeable epidermal phenotypes, whereas overexpression of Dhh and Shh caused complete loss of epidermal tissue renewal and epidermal progenitor cell hyperplasia as two distinct phenotypes. They also report overexpression of Shh, causing basal cell hyperplasia and the BCC phenotype [175].

### 2.7. MAPK/ERK Signaling

The MAP kinase or MEK (ERK kinase)/ERK (extracellular-signal-regulated kinase) pathway plays a significant role in a wide variety of multiple and divergent cellular functions, including cell proliferation, self-renewal, differentiation, apoptosis, and survival. MAPK/ERK signaling occurs downstream of various growth factors, cytokines, and hormone signaling, including FGF, EGF (epidermal growth factor), etc., and is well known for its role in cell proliferation. Three major groups of MAP kinases exist: the extracellular signal-regulated kinase (ERK) family, the p38 MAP kinase family, and the c-Jun NH2- terminal kinase (JNK) family.

ISC: One of the major driving forces for intestinal stem cell proliferation is the activation of the MEK/ERK pathway by the epidermal growth factor [176] (Figure 1). In vitro studies have shown that inhibition of MEK/ERK signaling suppresses ISC proliferation. The EGFR pathway, which is generally involved in the growth, survival, proliferation, and maintenance of cells, plays a vital role in regulating ISCs. Enteroblasts and ISCs express high levels of phosphorylated ERK, and disruptions in this signaling pathway cause proliferation defects and affect ISC survival [10]. Studies in *Drosophila* have found that EGFR signaling regulates ISC proliferation through MEK/ERK pathway [177]. The repression of RAS and ERK is sufficient to inhibit ISC proliferation [178]. Another major player in ISC proliferation, as discussed earlier, is Wnt signaling. The Wnt signaling interacts with the MEK/ERK pathway to suppress the ERK-mediated ISC proliferation and thus maintain the stem cell pool at the crypts. The authors have suggested that Wnt signaling maintains quiescent ISC by inhibiting the MAPK pathway [179]. Studies in *Drosophila* have also proved the role of the MAPK pathway in ISC proliferation. D-p38b MAPK is necessary for an age-associated increase of ISC proliferation and also for the differentiation of ISC into enterocytes. Age-related and oxidation stress-induced differentiation defects in ISC and progenitors are also associated with D-p38b MAPK [180].

In the planarian system, it has been found that egfr1 and nrg1 (putative EGF ligand) regulates intestinal progenitor cell differentiation. Silencing egfr1 and nrg1 impairs the differentiation of gamma neoblasts (gut lineage committed stem cells) into mature gastrodermal cells (phagocytes and goblet cells), without any effect on gamma neoblast specification. Silencing is also found to increase the number of gut progenitors as a consequence of differentiation failure [181,182]. 

NSC: Growth factor signaling through the MEK/ERK pathway influences the proliferation, differentiation, and migration of neural stem cells (Figure 2). A recent in vitro study has reported induction of neuronal differentiation upon MEK1 and 2 inhibition. RNAi-mediated inhibition of MEK2 increased neurogenesis, and overexpression of MEK2 conversely inhibited neurogenesis [183]. In the prenatal infection rat model, the intrauterine infection was found to increase the level of MAPK signaling pathway components, which acts to rescue infection mediated damage in the brain. Inhibition of MAPK signaling by small molecule MEK inhibitor caused a diminished rescue effect by MAPK signaling indicated by aggravated hippocampal neuronal apoptosis, decreased cell proliferation and differentiation, decreased neurogenesis, and impaired cognitive performance [184]. Several reports have identified MAPK/ERK signaling as a positive regulator of neural stem cell proliferation [185,186,187,188]. Neurotransmitter dopamine and amino acid taurine are also reported to induce proliferation of neural stem/progenitor cells mice through ERK1/2 pathways [189,190]. Other than these, MAPK signaling also acts downstream of signals that induce Neural stem/progenitor migration towards the site of injury. Inhibition of ERK1/2 phosphorylation inhibits NSC migration. Using p38 small molecule inhibitors, it was demonstrated that the endogenous p38 is involved in NSC migration. Introducing wild-type p38 protein into inhibitor-treated cells significantly increased adult NPC migration without any changes in cell survival or differentiation. Inhibition of MEK also reduces migratory properties of human neural progenitors due to inhibition of ERK1/2 phosphorylation. An ERK-independent control of migration also exists, which acts through PKC and EGFR [191,192]. 

The MEK/ERK pathway is also reported to play a role in regulating gliogenesis in mice. Deleting Mek1/2 in radial progenitors (capable of giving rise to neurons and glia) inhibits their transition to glial fate with reduced glial progenitors in the cortex. These mutants did not exhibit any defect in the formation of neurons. Conversely, overexpression of a constitutively active MEK1 induces precocious glial specification with increased astrocyte numbers, along with reduced neuronal numbers in the cortex [193]. This report indicates the role of MEK1/2 as a fate-switch controlling the glial or neuronal fate within cortical radial progenitors. Other in vitro data also indicate the role of ERK1/2 in astrocyte proliferation. Activation of ERK1/2 induced by SDF-1α was found to increase proliferation in rat type-1 astrocytes [194].

The role of the EGFR signaling pathway in regulating stem cell dynamics in planarians has been recently thoroughly studied and reviewed [181]. In planarians, egr4 (putative target of EGFR3) regulates the neoblast differentiation and anterior regeneration. Egr-4 is mainly expressed in the CNS and regulates head regeneration and brain development and is necessary for the early renewal of brain primordia. Its silencing results in aberrant development of cephalic ganglia and photoreceptors truncated ventral nerve cords and reduced neuronal subtypes, including octopaminergic neurons and dopaminergic neurons [181,195]. The intracellular cascades activated by different EGF receptors in planarians are largely unknown. However, as ERK inhibition produces similar phenotypes to those described after egfr-3 RNAi, we may speculate that egfr-3 might be exerting its activity via the ERK pathway [181,196]. In addition to this function, egfr-3 has been shown to regulate asymmetric cell division in planarians through its putative ligand neuregulin-7 (nrg-7). The ratio of asymmetric to symmetric cell divisions decreases significantly in egfr-3(RNAi) worms [197]. The nou-darake (ndk), a gene encoding a fibroblast growth factor receptor (FGFR)-like molecule, is also identified and characterized in planarians. Knockdown of the ndk in whole animals results in the induction of ectopic brain tissues throughout the body. The ablation of stem cells by irradiation inhibits the development of this phenotype, implicating the involvement of stem cells in ectopic brain formation [198]. In planarians, ERK is required for the differentiation of stem cells into multiple cell lineages. Inactivation of ERK signaling by treatment with U0126 (a selective inhibitor of both MEK1 and MEK2) causes severe defects in both head and tail regeneration [196]. During regeneration, mkpA, a planarian mitogen-activated protein kinase (MAPK) phosphatase-related gene, is specifically expressed in the blastema cells. The mkpA RNAi can rescue these regeneration defects caused by U0126. Hence, it is suggested that ERK signaling plays an instructive role in the cell fate decisions of blastema cells regarding whether to differentiate or not by inducing mkpA as a negative regulator of ERK signaling during planarian regeneration [196].

HSC: In hematopoietic stem cells, the MAP kinase pathway studies mostly concern the pathological conditions and malignancies and are performed in vitro, using isolated stem cells. Several studies indicate the role of MAP kinase signaling in controlling normal and malignant hematopoiesis [199,200]. Several of the cytokines necessary for the normal proliferation and survival of HSCs, like erythropoietin, IL3, GM-CSF, stem cell factor (SCF) etc., were found to activate different MAP kinases [199,201]. An In Vivo study using mice doubly-deficient in ERK1 and ERK2 showed rapid decline of HSCs and immature progenitors in a cell-autonomous manner. The absence of both kinases causes bone marrow aplasia, leukopenia, anemia, and early lethality in mice [202]. In Vivo evidence from Atm-/- mice also shows that p38 MAPK is activated in response to increased ROS (reactive oxygen species) levels. ROS activate MAPK, causing defects in the HSC quiescence and repopulation capacity. Moreover, inhibition of p38 MAPK was able to rescue this phenotype [203].

EpSc: The classical MEK/ERK pathway plays an important role in controlling the proliferation and differentiation in the epidermis (Figure 4). Activation of this pathway generally promotes proliferation in the epidermis and inhibits differentiation. However, there are contradicting in vitro results from different groups. Loss and gain of function studies using Ras/Raf/Mek have shown that this pathway is necessary to maintain the homeostatic proliferation and self-renewal divisions in the epidermis. The gain of function studies have reported hyperproliferation in the epidermis, whereas loss of function induces differentiation and hypoproliferation [204,205]. Inducible expression of MEK1 in mice epidermis causes epidermal hyperplasia and an increase in the phosphorylated ERK1/2. However, this phenotype was not observed upon induction of MEK2 [206]. Similar hyperproliferative phenotypes with reduced differentiation are observed upon induction of RAS in mice epidermis as well (Figure 4C). This RAS activation is associated with an increase in β1 and β4 epidermal progenitor markers [207]. A very recent report using live imaging in mice epidermis also suggests that basal keratinocytes had higher levels of ERK activity compared to more differentiated suprabasal layers. They have shown that the pulsative levels of ERK activity are different from the basal active levels and are regulated independently. This pulsatile ERK activity controls the epidermal stem cell fate. Pulsative downregulation of ERK in mouse epidermis was associated with differentiation, and these changes in levels might be the switch that controls division and differentiation states [208]. Another very recent study in mice reports reduced thickness of epidermal layers, delayed hair follicle development, and dehydration states in tumor suppressor WW domain-containing oxidoreductase (WWOX)-deficient mice, which in turn is associated with reduced levels of MEK/ERK signaling in those WWOX-deficient keratinocytes [209]. This mutant mouse also showed increased cell proliferation in the junctional zone progenitors of hair follicles but a reduced number of hair follicle stem cells at the bulge.

## 3. Concluding Remarks

ASC dynamics need precise regulation to avoid any compromise with tissue health. Multiple extrinsic and intrinsic factors influence ASC behavior through genetic and epigenetic regulations. Detailed insights into these regulations would help us understand various pathophysiological processes such as regeneration, degenerative diseases, and ageing. It is well known that several microenvironmental factors influence ASC behavior. Therefore, In Vivo investigations are required to observe the multidimensional behaviors of ASCs in their natural microenvironment that permits their cross-talks with other cell types/ extracellular matrix. However, the accessibility of ASCs is a significant challenge for their In Vivo observations and manipulations. Very few animal models provide easy access to their ASCs for In Vivo investigations. One such model system is the planarians. Planarians possess a pool of ASCs distributed throughout their body and we can conveniently study their dynamics In Vivo inside the animals. Planarians would particularly be useful for studying dynamics of epidermal stem cells, intestinal stem cells, and neural stem cells but not for hair bulge stem cells or hematopoietic stem cells. Molecular techniques to study these animals have evolved significantly in the past two decades. This makes planarians a suitable model for studying ASC behaviors, and further investigations hold enormous promise for contributing novel insights into the current understanding of the regulation of ASC dynamics.

## Data Availability

Not applicable.
